# Comparative Dissection of Three Giant Genomes: *Allium cepa*, *Allium sativum*, and *Allium ursinum*

**DOI:** 10.3390/ijms20030733

**Published:** 2019-02-09

**Authors:** Vratislav Peška, Terezie Mandáková, Veronika Ihradská, Jiří Fajkus

**Affiliations:** 1 Institute of Biophysics, The Czech Academy of Sciences, Královopolská 135, 612 65 Brno, Czech Republic; ihradska.v@gmail.com; 2 Mendel Centre for Plant Genomics and Proteomics, CEITEC, Masaryk University, Kamenice 5, CZ-62500 Brno, Czech Republic; Terezie.Mandakova@ceitec.muni.cz

**Keywords:** *Allium*, plant genome, repeats, retrotransposon, satellite, telomere, rDNA, RepeatExplorer, TAREAN

## Abstract

Knowledge of the fascinating world of DNA repeats is continuously being enriched by newly identified elements and their hypothetical or well-established biological relevance. Genomic approaches can be used for comparative studies of major repeats in any group of genomes, regardless of their size and complexity. Such studies are particularly fruitful in large genomes, and useful mainly in crop plants where they provide a rich source of molecular markers or information on indispensable genomic components (e.g., telomeres, centromeres, or ribosomal RNA genes). Surprisingly, in *Allium* species, a comprehensive comparative study of repeats is lacking. Here we provide such a study of two economically important species, *Allium cepa* (onion), and *A. sativum* (garlic), and their distantly related *A. ursinum* (wild garlic). We present an overview and classification of major repeats in these species and have paid specific attention to sequence conservation and copy numbers of major representatives in each type of repeat, including retrotransposons, rDNA, or newly identified satellite sequences. Prevailing repeats in all three studied species belonged to Ty3/gypsy elements, however they significantly diverged and we did not detect them in common clusters in comparative analysis. Actually, only a low number of clusters was shared by all three species. Such conserved repeats were for example 5S and 45S rDNA genes and surprisingly a specific and quite rare Ty1/copia lineage. Species-specific long satellites were found mainly in *A. cepa* and *A. sativum*. We also show in situ localization of selected repeats that could potentially be applicable as chromosomal markers, e.g., in interspecific breeding.

## 1. Introduction

The genus *Allium* (Amaryllidaceae) includes more than 800 species, making it one of the largest monocotyledonous genera. For the purpose of this study we selected the two species which, according to the Food and Agriculture Organization of the United Nations (FAO), make the largest contribution to food production, *A. cepa* and *A. sativum*, (onion and garlic), and we also included one distant relative, *A. ursinum* (wild garlic), which is of only marginal economic importance, but is interesting for several other reasons. Firstly, it grows relatively abundantly and is native to Europe and Asia. Secondly, *A. ursinum* belongs to the oldest evolutionary clade in *Allium*. The other two selected species, *A. cepa* and *A. sativum*, belong to the youngest evolutionary line [1].

*Allium* genomes belong to the group of giant plant genomes: *A. cepa* has 1C=16.75 pg [2], *A. sativum* has more or less the same genome size 1C = 16.25 pg [3], but *A. ursinum* has almost twice as large a genome 1C = 31.45 pg in comparison with the previous two [4]. *A. cepa* and *A. sativum* represent members of the genus with the most common chromosome number *x* = 8, but other numbers (e.g., *x* = 7, 9, 10, 11) and variability in ploidy also occur (reviewed in [1]). *A. ursinum* is an example with the chromosome number *x* = 7. 

The *Allium* genome assembly has not been released so far. There are several genomic strategies on how to prepare an informative repeatome of an as yet unassembled genome, but most of these need some reference genome of a closely related species or general repeat database at least, and NGS data with high genomic coverage. On the other hand, identification of major repeats *de novo* using RepeatExplorer, TAREAN and Tandem repeats finder is independent on any reference sequence [5,6,7]. 

Among repetitive elements, special attention is paid to ribosomal RNAs (rRNAs) and their genes (rDNAs) due to the essential importance of rRNA in proteosynthesis. These remnants of the RNA-world are relatively highly conserved and their genes are present in every cellular genome (reviewed in [8]). However, the structure and organization of rDNA may differ [9]. 45S rDNA codes for 18S, 5.8S and 25S rRNA, while 5S rDNA codes for 5S rRNA. Both loci may exist either separately as tandemly repeated units, or in a linked arrangement where a single unit of 5S rDNA is inside the intergenic spacer between 45S rDNA units. Physical linkage between 45S and 5S rDNA in plants has been described in some early diverging taxa of mosses, algae and ferns [10,11], while a separate arrangement is typical for most land plants. Exceptions were discovered, e.g., in Asteraceae [12] and gymnosperms [13]. The number of loci possessing 45S and 5S varies from a single one to several tens per haploid genome [14]. Their number is usually species-specific, however, exceptions with hypervariability and polymorphisms in the rDNA signals were described in *Anacyclus* (Asteraceae) [15]. 

Most *Allium* species studied have a low number of rDNA clusters per genome, except for *A. ursinum*, where amplification of 45S rDNA was observed [16]. One strong and one very weak signal of 45S rDNA per haploid genome were detected in *A. fistulosum* (*n* = 8), while two strong signals of 45S rDNA clusters per haploid genome were described in *A. cepa* [17]. In *A. wakegi* (*n* = 8), a natural allodiploid between *A. cepa* and *A. fistulosum*, three signals per haploid genome were detected [18]. Genbank contains numerous accessions with short partial 45S rDNA sequences from *Allium*, e.g., partial gene sequences and intergenic spacers from *A. ursinum* (HF934582; 456 nt, KM103427; 1174 nt, and KX167936; 470 nt). Two notably long sequences represent clones containing either complete 45S rDNA unit sequences (KM117265; 10,621 nt) or partial sequences of 25S and 18S rRNA genes and the spacer between them from *A. cepa* (EU256494; 6117 nt). Regarding 5S rDNA, one locus was observed e.g., in *A. fistulosum*, two distinct loci of 5S rDNA in *A. cepa*, and three in *A. sativum* [19,20]. Numerous 5S rDNA sequences are publicly available for several *Allium* species, e.g., for *A. cepa* (Genbank AF101244; 352 nt and KM117264; 522 nt) and *A. sativum* (AF101249; 603 nt).

Centromeres are nucleoprotein structures responsible for proper segregation of chromosomes in mitosis and meiosis, and they are usually species-specific in their sequences. The characterization of centromere elements *de novo* is well described in [21]. The method is based on Chip-Seq using antibodies specific for the centromere histone variant CenH3. The only centromere chromatin immunoprecipitated sequences from *Allium* are from *A. fistulosum* (GenBank AB735740, AB735741, AB735743, AB735742, AB735744; 144–376 nt) [22].

Telomeres are chromatin structures forming the ends of linear chromosomes. Their function is to protect the natural ends and distinguish them from DNA breaks. The telomere sequence is usually formed by a minisatellite with a motif that can be summarized as (T*_x_*A*_y_*G*_z_*)*_n_*. Recently we demonstrated that an unusual telomere repeat (TTATGGGCTCGG)_n_ forms telomeres in 11 *Allium* species [16], suggesting that the sequence is common across the genus.

Long tandem repeats were among the first characterized in *A. cepa*. It was shown that approximately 4% of the *A. cepa* genome is composed of a satellite with a unit of 375 bp and it localizes near all chromosomal termini except the ends occupied by nucleolus organizer regions (NORs). Three variants of this satellite were published, ACSAT1, ACSAT2, and ACSAT3 (GenBank X02572; 372 nt), differing in several single nucleotide positions and short insertions and deletions [23]. Much higher variability was found in more detailed studies, when sequence and length variants were described, including, e.g., a 314 bp long repeat called pAc074 (GenBank AF227152; 314 nt), [24]. This showed a similar terminal localization as ACSATs. Similar satellites were detected and characterized in closely related *Allium* species, such as *A. fistulosum* (AFISAT), which is 378–380 bp long and it has 82% homology to ACSAT. Furthermore, from *A. roylei*, AROSAT (GenBank: Z69035), *A. oschaninii*, AOSAT (GenBank: Z69034), *A. altaicum*, AALSAT (GenBank: Z69033), and from *A. galanthum*, AGASAT were described (GenBank: Z69036) [25]. It was estimated that 2.8 million copies of AFISAT exist per haploid *A. fistulosum* genome, which represents a genomic proportion of 4.5% [26]. An original model of a simple tandem array built only from satellites has been updated with information about inversions of its repeat units, interruptions by microsatellites, and Ty1/copia elements [27].

Recently, two new tandem repeats with specific chromosomal positions were described in *A. fistulosum*. The first one was called “Heterochromatin-associated *Allium* tandem repeat from CL58” (HAT58, KX137121; 65 nt). Species-specific HAT58 has a unit 65 nt long and it localizes to chromosomes 6, 7, and 8. It occurs as a polymorphic region on chromosome 7. The second repeat, “periCentromeric *Allium* tandem repeat from CL36” (CAT36, KX137122; 197 nt) localizes to pericentromeres of chromosomes 5 and 6. These two repeats represent 0.25% of the *A. fistulosum* genome. It was estimated that there are 160,000 copies of HAT58 and 93,000 copies of CAT36 per haploid genome. [28]

Mobile elements represent another important class of repeats in *Allium* genomes. Heterogeneous retrotransposons belonging to the Ty1/copia group were estimated to represent roughly 3% of the *A. cepa* genome, (100,000–200,000 copies per haploid genome). Ty1/copia elements were shown to be distributed all over the *A. cepa* chromosomes, but they were enriched in the terminal heterochromatin. A similar finding was obtained in the case of EnSpm-like DNA transposons [29,30]. An enrichment of Ty3/gypsy retrotransposons in the centromeres was observed in *A. cepa* and *A. fistulosum*, using a probe derived from a genomic clone (GenBank ET645811; 910 nt). The total number of these centromere-enriched retrotransposons was estimated at 26,000 copies per haploid genome, i.e., 0.89% in terms of genomic proportion. The authors of this study hypothesized that centromeric chromatin is a safe environment due to its reduced rate of recombination and thus a lower risk of removal of the element from the genome [31].

Overall, patterns of diverse types of repeats may reflect processes involved in speciation and karyotype evolution. Furthermore, knowledge of repeatomes can be applied to breeding strategies in respective crop species. In this study, we perform a comparative description of repeats in three *Allium* species using a genome assembly-independent approach, and provide a repeat database with information on major short and long tandems, mobile elements, and plastid DNA. 

## 2. Results

### 2.1. Comparative Analysis of Short Tandem Repeat Motifs with A Length Between Three and 52 bp

**Figure 1:**
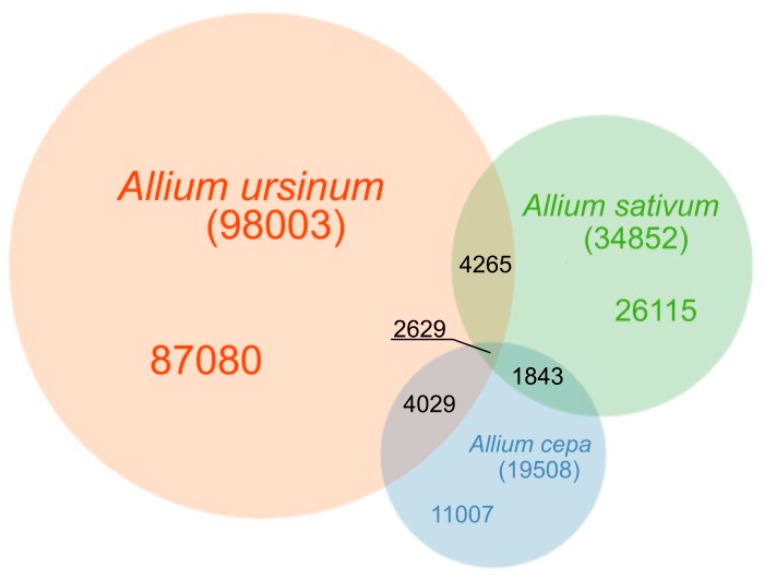
Venn diagram of detected short tandem motifs in *A. cepa*, *A. sativum* and *A. ursinum*. The diagram shows how many distinct motifs were identified in the three species and how many of them are shared. However, it represents a total sequence variability of detected motifs rather than genome proportions of these motifs, because the largest variability in the sequence of the motifs comes from very rare short tandem repeats. In the shortest motifs predominantly AT rich sequences are detected. This trend of excluding GC rich sequences in tandems is not so obvious in the case of the longer motifs we obtained.

In this part of the article, we describe results related to tandem repeats with a motif length between three and 52 bp, that are difficult to analyze in RepeatExplorer because of their low complexity and abundancy in the genome. Considering all tested species, we identified nearly 137 thousand (136,968; Table S1) distinct short tandem motifs (STM) in Tandem Repeats Finder–Tandem Repeats Merger (TRFi–TRM) raw output. The number of motifs detected and their genomic proportion differed in all species studied (Figure 1). The lowest number of distinct motifs and their genomic proportion was in *A. cepa*, where 19,508 motifs, representing 0.9% of the genome were detected (Table S2). In *A. sativum* ca. twice the number of motifs (34,852) was detected (Table S3). We estimate that this represented 2.3% of the genome. The genome of *A. ursinum* was similar to *A. sativum* in total genomic proportion of short tandem repeats (2.4%) but differed considerably in the variability of these motifs (98,003 distinct motifs) (Table S4). 

**Figure 2:**
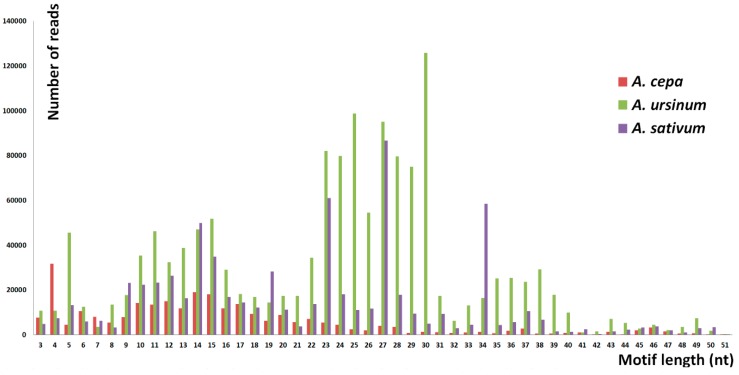
The abundancy of short tandem motifs. The chart shows a distribution of the detected reads with tandem motifs of a specific length. Height of the bars represents the number of reads and the position corresponds to the increasing lengths of the motifs. The most frequent lengths are 4 nt in *A cepa*, 27 nt in *A. sativum* and 30 nt in *A. ursinum*. The majority of repeats is not distributed equally in the three species. *A. cepa* has most of the tandems around 9–18 nt, *A. ursinum* has a majority around 23–30 nt, and *A. sativum* shows peaks of 5, 9–15, 19, 23, 27, and 34-nt long motifs.

**Figure 3:**
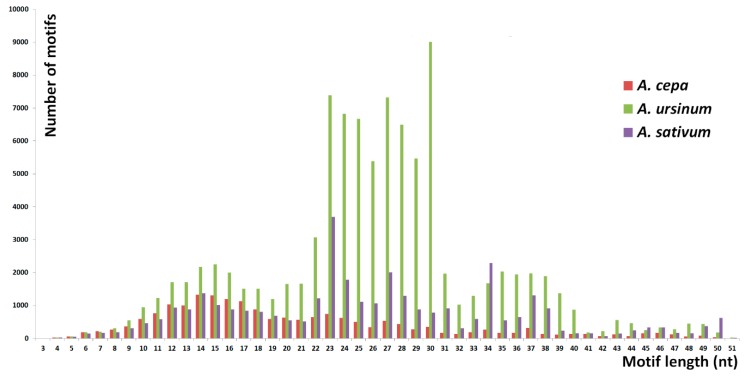
The number of short tandem motifs. This chart shows a distribution of the number of distinct motifs with the same length. Unlike the abundancy of tandem lengths (Figure 2), the tandems with lengths up to around 20 show similar profiles in all three species. Most of the differences are hidden in lengths 20–40 nt. Particularly in *A. ursinum*, there is a very large increase in the number of distinct motifs around lengths of 23–30 nt. In the case of *A. sativum*, several peaks are detected, e.g., in lengths of 23, 27, and 34 nt.

The number of specific and shared motifs in species studied is depicted in Figure 1. The three species differed in the lengths of tandem motifs that represented the majority of repeats detected. In *A. cepa*, the largest proportion was formed by 9–18 nt long motifs. Outstanding high peaks occurred at 4 nt (Figure 2). Unlike the 4 nt repeats, in *A. cepa*, the area from 9–18 nt included a lot of variability in motif sequences (Figure 3). The category of 4 nt tandems includes the most abundant *A. cepa* short tandem repeat (TTAA)*_n_* (Figure S1). In *A. ursinum*, the majority of motifs was spread around lengths from 23 to 30 nt (Figure 2) and within this range, even the variability (number of distinct motifs with a specific length) was enormous (Figure 3). Other local maxima in *A. ursinum* were around lengths of 5, 10–16, and 34–40 nt (Figure 2). In *A. sativum*, notable peaks occurred around 5, 9–15, 19, 23, 27, and 34 nt long motifs (Figure 2 and Figure 3). Generally, there was a remarkable difference between the distribution of abundancy and the number of motifs up to a length of ca. 10 nt (microsatellite and minisatellite tandems), as can be seen particularly in *A. cepa* at 4 nt, and at 5 nt in *A. ursinum* and *A. sativum* (Figure 2 and Figure 3). 

The most abundant length of 27 nt corresponded to the most abundant specific sequence (AATCGTTTTCAATTCAATTCATTTCGA)*_n_* in *A. sativum*, while in the other two species the most abundant sequences were much shorter. The motif (TTAA)*_n_* was the most abundant short tandem repeat in *A. cepa*. In *A. ursinum*, the most frequent motif was (ATCCG)*_n_*. (Figure S1). Frequencies and sequences of other prevalent motifs according their sequence are listed in Supplementary Tables S1–4.

### 2.2. Telomere Sequence

The telomere sequence (CTCGGTTATGGG)*_n_* was detected here as one of the most abundant minisatellite motifs with a unit length of 12 nt in all three species: In *A. cepa*, 745 telomere reads were detected out of 30 million. This corresponds to 0.00248% (406.224 kb per haploid genome, 25.389 kb per telomere on average). In *A. sativum*, 1519 reads in 30 million total reads. This corresponds to 0.00506% (804.563 kb per haploid genome, 50.285 kb per telomere on average). Finally, in *A. ursinum*, 2485 reads in 60 million total reads, corresponding to 0.00414% (1,273.976 kb per haploid genome, 90.998 kb per telomere on average).

Reads with ancestral telomere motifs, (TTTAGGG)*_n_* and (TTAGGG)*_n_*, were identified in all three species. According to the number of reads detected, the sequences constituted a scant genomic proportion, corresponding to a single copy gene or barely a few thousand base pairs, which is congruent with previous observations [32,33]. 

### 2.3. Comparative Clustering of Interspersed and Long Tandem Repeats

#### 2.3.1. Summary

**Figure 4:**
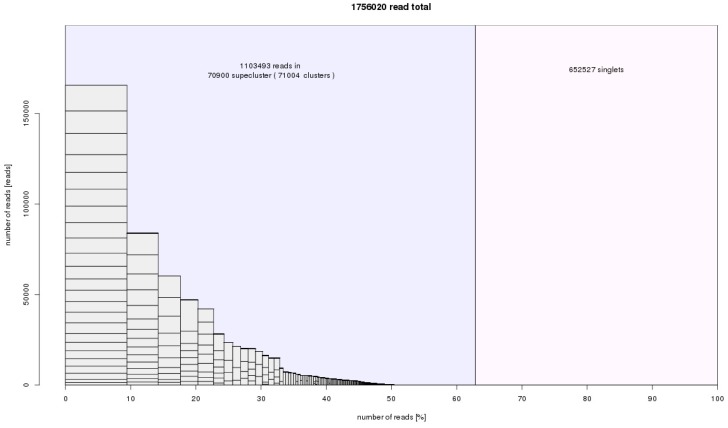
RepeatExplorer histogram summary of superclusters, clusters and non-clustered reads. The input was 1,756,020 reads from *A. cepa*, *A. ursinum*, and *A. sativum*. The number of reads from each species was normalized according to genome size. We obtained 70,900 superclusters representing groups of repetitive elements. The genomic proportion of these identified repeats was 63%. The non-clustered reads, which represent 27% of genome, are low copy and single copy sequences that cannot be characterised in RepeatExplorer analysis. This statistic is slightly distorted by plastid sequences that also form separate clusters. They are not further counted after cluster annotation (Table 1).

From a total input of 1,800,000 reads (151 nt-long paired end reads), 1,756,020 sequences were analyzed in RepeatExplorer and TAREAN. In the case of *A. cepa*, 146,302, 146,482, and 146,308 reads were analyzed (datasets S1–3). In *A. ursinum*, 292,614, 292,752, and 292,474 reads were analyzed (datasets S4–6). Finally, in *A. sativum*, 146,366, 146,318, and 146,404 were analyzed (datasets S7–9). The number of reads analyzed corresponded to approximately the same genomic coverage (0.004×, ~0.4% of the genome) for all three species in the comparative clustering RepeatExplorer and TAREAN analysis. Differences between the numbers of input and analyzed reads were caused by the RepeatExplorer automatic optimization of input considering the complexity of genomes and the computational capacity of the server. Approximately 63% of input (1,103,493) reads were grouped into 71,004 clusters, representing prevailing repeat families in the *Allium* species studied. The rest, singlets, are reads that were not in any cluster, representing low copy repeats and single copy sequences. Thanks to the experimental setup in the paired end reads whole genome shotgun sequencing, we were able to connect clusters into so called superclusters. In many cases, superclusters represent a prediction of full-length repeat consensus and variability, particularly in the most abundant repeats (Figure 4). 

The clusters were joined to superclusters based on information about mates from paired end reads in two steps. The first supercluster formation was done automatically by the pipeline and the second step was a manual correction and fusion of separate superclusters, obviously originating from the same element. For example, plastid clusters formed mostly separate superclusters before manual correction and joining them to supercluster number 40 (SC40) (Figure S2). In this way we obtained a total of 117 superclusters for all three species. Annotation was done based on RepeatExplorer automatic prediction and manual modification taking into account the information on a similarity and paired-end mates from well characterized clusters.

#### 2.3.2. RepeatExplorer

Most of the superclusters were disproportional in the species representation (Table S5). On the other hand, superclusters corresponding to 45S, 5S rDNA and, interestingly, also to the retroelement Ty1/copia/TAR were obviously shared among all three species. The other superclusters were usually rather species-specific even if they represented repeats from the same lineage (e.g., Ty3/gypsy/Tekay), and the reads from related species were detected in the same supercluster with a frequency several orders of magnitude lower. The comparison of transposons was done at the level of repeat element class and lineage (Table 1).

##### DNA Transposons

The only DNA transposon detected in our comparative analysis from *Allium* was EnSpm/CACTA. It was slightly expanded in *A. ursinum* compared to *A. cepa* and *A. sativum.*

##### Ty3/Gypsy Retrotransposons

Four lineages of Ty3/gypsy retrotransposons were detected in our data, chromovirus-like Tekay and CRM, and non-chromovirus-like Athila and TatV. Tekay was higher in genomic proportion in all three species. However we detected less of it in *A. cepa* (24.86%) compared to the other two species (ca. 45.51% and 47.88%). The second most prevalent repeat, TatV lineage of Ty3/gypsy, was tenfold less abundant in *A. cepa* and *A. ursinum*, and 40 times less abundant in *A. sativum* in comparison to Tekay. The rare, although consistently found Athila element, was discovered in all three species. In contrast, CRM from *A. cepa* (0.66%) was rarely detected in *A. ursinum* and *A. sativum*. 

##### Ty1/Copia Retrotransposons

Ty1/copia elements were much less abundant in *Allium* than gypsy retrotransposons, but they were interesting in several ways. For example, the Ty1/copia/TAR element was well conserved. A sublineage from a single cluster (CL111) was formed by reads from all three species. This cluster included all protein coding domains of the element (Figure S3). A consensus sequence of this element is shown in supplementary file (Supplementary File S1), and more details on this element are given in the TAREAN results (Section 2.3.3.). Another Ty1/copia element, Tork, was detected only in *A. ursinum*. The last detected Ty1/copia retrotransposon belongs to the lineage SIRE. In all three species it formed only tiny genomic proportions.

##### Terminal-Repeat Retrotransposons in Miniature

A tiny amount of potential Terminal-repeat Retrotransposons In Miniature (TRIM) was detected in all three species. They formed separate clusters with contigs up to several hundreds of base pairs that did not code for any protein but did contain a PBS (primer binding site) and obvious insertion border between the element and random genomic sequence of the target site. These characteristics are typical for TRIM [34].

##### Non-LTR Retrotransposons

Non-LTR retroelements were rather rare in the *Allium* genomes. All detected LINE variants, including its probable daughter group of elements, SINE, represented less than 1% of the genome in each of the species studied. Slightly higher levels of LINEs and SINEs were detected in *A. ursinum*.

##### Tandem Repeats

We observed an interesting trend in the occurrence of long satellite DNA in the plants studied. In *A. ursinum*, with the largest genome, we detected only a 0.17% genomic proportion of satellite DNA. *A. sativum* had almost twice the genomic proportion of satellite DNA (0.31%) and *A. cepa*, more distant to *A. ursinum*, had almost ten times more satellite DNA (1.68%) than *A. ursinum*.

The 45S rDNA appears to be the most evenly distributed repeat among the species studied. Its genomic proportion was 0.38% in *A. cepa*, 0.33% in *A. sativum*, and 0.26% in *A. ursinum*. The 5S rDNA cluster content was less even compared to 45S. This was due to the lower coverage and incomplete assembly of the whole repeat unit in *A. ursinum* and sequence variability in the spacer of *A. sativum*. 

Tandem sequences were also analyzed using a parallel tool with a more efficient algorithm for graph clustering–Tandem Repeat Analyzer (TAREAN, see the next paragraph). Consensus sequences of long tandem repeats are shown in Supplementary File S1, raw RepeatExplorer output from comparative analysis can be seen in Supplementary Files S2–S4.

#### 2.3.3. TAREAN: TAndem REpeat ANalyzer

The RepeatExplorer pipeline was recently upgraded with a tool called TAREAN, which is targeted to tandem repeats analysis, particularly larger satellites than the read length and also interspersed repeats with a circular graphical shape, e.g., LTR retrotransposons [6]. In the case of our three *Allium* species, we detected four species-specific clusters corresponding to satellite sequences in comparative graph-based clustering. Clusters shared by all three species corresponded to LTR elements Ty1/copia TAR and 5S rDNA.

##### AcepSAT750: Allium cepa Satellite with a 750 bp Long Consensus Monomer (CL180)

**Figure 5:**
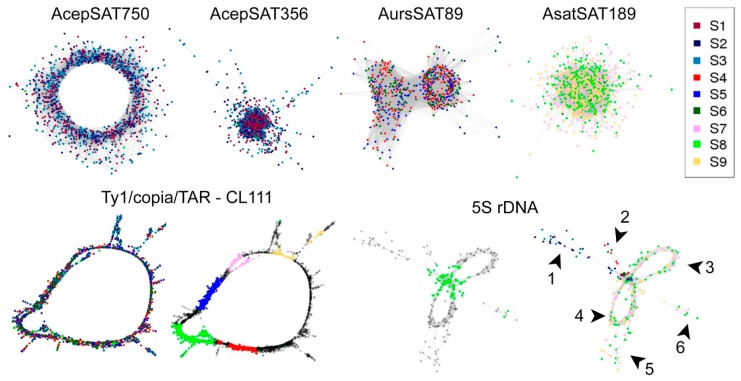
Results of graph-based clustering in TAREAN comparative analysis. AcepSAT750 has a ring-shaped cluster of exclusively *A. cepa* reads. Repeats with shorter monomer lengths, AcepSAT356, AursSAT789, and AsatSAT189 have more compact graphs. Surprisingly, one Ty1/copia/TAR sublineage forms a graph shared by all three species (genes in a typical order for copia-like elements: GAG—yellow; PROT—pink; INT—blue; RT—green; RH—red). Consensus of these repeats is in the separate supplementary file File S1. The graph of 5S rDNA presents information about the reads containing 5S rRNA gene—green dots, and species-specific intergenic spacers are labelled by arrowheads and numbers (1—*A. cepa*; 2—*A. ursinum*; 3–6—*A. sativum*).

We detected a putative satellite with a 750 bp long consensus unit in the *A. cepa* genome. The actual size of the cluster reached 1221 reads that were almost exclusively from *A. cepa* (Figure 5; AcepSAT750). This number of reads represented 0.28% of the *A. cepa* genome (the total number of reads from *A. cepa* analyzed in comparative clustering was 439,092). ACSAT750 was predicted with a high confidence that was based on several parameters of its cluster and graph analysis, such as connected component (index C) and pair completeness (index P) [6]. The consensus sequence of this repeat is shown in Supplementary File S1. In addition to the consensus sequence, representing the major variant, numerous monomers with different lengths were predicted (409–750 nt, Supplementary File S2). 

##### AcepSAT356: Allium cepa Satellite with a 356 bp Long Consensus Monomer (CL50)

The other species-specific satellite monomer was 356 nt in length. Its actual cluster size was 5875 reads (Figure 5; AcepSAT356). This number of reads represented 1.3% of the *A. cepa* genome, 217Mb per haploid genome in total. This satellite was predicted with a low confidence. The consensus sequence of this repeat is shown in Supplementary File S1. In addition to the consensus sequence, representing the major variant, numerous monomers of different lengths were predicted (21–378 nt, Supplementary File S2). The 21 nt-long monomer consensus comprises reads with sequence homology to a nested tandem repeat inside of AcepSAT356.

##### AsatSAT189: Allium sativum Satellite with a 189 bp long Consensus Monomer (CL172)

An *A. sativum* species specific satellite-like candidate was comprised of a monomer of length 189 nt. Its real cluster size was 1300 reads. This number of reads represents 0.3% of the *A. sativum* genome, 48 Mb per haploid genome in total. This satellite was predicted with a low confidence. The consensus sequence of this repeat is in supplementary file S1. In addition to the consensus representing the major variant, several monomers with differing lengths were predicted (20–379 nt, Supplementary File S2). 

##### AursSAT89: Allium ursinum Satellite with a 89 bp Long Consensus Monomer (CL237)

The *A. ursinum* species specific satellite-like monomer sequence was 89 nt in length. Its cluster real size was 510 reads. This number of reads represents 0.06% of the *A. ursinum* genome, 19 Mb per haploid genome in total. This satellite was predicted with a low confidence. The consensus sequence of this repeat is in Supplementary File S1. In addition to the consensus representing the major variant, several monomers with different lengths were predicted (38–379 nt, Supplementary File S2). 

##### 5. S rDNA (CL274)

As with the majority of plants, *Allium* has 5S rDNA arranged in tandems and the cluster graph has similar characteristics such as general satellite sequences. Conserved regions of 5S clusters corresponding to a 5S rRNA gene are common for all three species (green arrow and dots in cluster of 5S rDNA in Figure 5. Species specific spacers differ in sequence and length (numbers 1–6 in cluster of 5S rDNA in Figure 5). Species specific contigs for the 5S rDNA are listed in Supplementary File S1.

##### LTR Ty1/Copia/TAR (CL111)

TAREAN can also predict and analyze other repeats with a circular cluster graph like LTR elements. In the case of the *Allium* species examined, an LTR Ty1/copia/TAR element was detected. Its consensus sequence was 5417 nt long and was present in all three species. According to comparative analysis, it constituted around 30 Mb (0.2% genomic proportion) in *A. cepa*, 76Mb (0.2% genomic proportion) in *A. ursinum*, and 4.8Mb (0.03% genomic proportion) in *A. sativum* per haploid genome. The consensus sequence of this repeat is shown in supplementary file S1. In addition to the consensus representing the major variant containing all Ty1/copia-like domains, several monomers with different lengths were predicted (3–5457 nt, supplementary File S2). The monomer consensus units with short lengths e.g., 3 nt, had sequence homology to nested tandem repeats inside of this family LTR Ty1/copia /TAR element. (Figure S3 and a graph in Figure 5).

### 2.4. Independent RepeatExplorer, TAREAN Analysis, and Separate Repeat Annotation for Each of the Species

**Figure 6:**
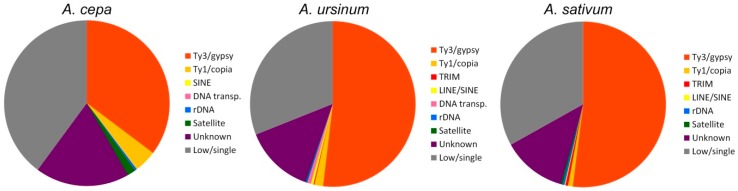
Pie graphs of repeats detected in separate analyzes show comparison of repeat elements according to their repeat classification. Purple sections represent the proportion of repetitive sequences in large clusters that either stayed unannotated or small clusters that were not analyzed in detail. Grey sections represent low and single copy sequences that stayed as non-clustered single reads.

Independent clustering with a higher coverage was done to annotate species-specific sequences and also repeats that were shared among the species studied. General statistics of the abundancy of repeat lineages detected in separate clustering and annotations is shown in Figure 6. Unlike comparative clustering, more Ty1/copia lineages (Bianca, Ikeros, and Angela) were found by separate clustering. Generally, the higher coverage enabled deeper annotation that led to a slight increase in the genomic proportion identified, e.g., in the Ty3/gypsy/Athila group (Table 1 and Table 2). On the other hand, Ty3/gypsy/CRM was found only in *A. cepa* and not in other species in this way. 

In the case of *A. cepa*, the RepeatExplorer output consisted of 52,485 clusters, 17 of which represented plastid DNA (File S5). Clusters without plastid sequences were repeats representing ca. 60% of the genome. Due to rational simplification, only the large (CL1–CL223) clusters, which together represent 45.66% of the genome, were analyzed in detail and annotated. The remaining small and numerous clusters (14.45% of genome) were not further processed. In the case of *A. ursinum*, the RepeatExplorer output consisted of 48,182 clusters, from which 16 clusters represented plastid DNA (File S6). Large clusters (CL1-CL298) represented 58.36% of the genome. In the case of *A. sativum*, the RepeatExplorer output consisted of 26,094 clusters, from which 20 clusters represented plastid DNA (File S7). The largest 223 clusters represented 55.84% of the genome. The majority of clusters analyzed in detail were annotated. Only 1.48–3.93% of clusters analyzed in detail remain unclassified. Nevertheless, a much higher content (10.6–14.45% genomic proportion) remain uncharacterized in small clusters that were not analyzed in more detail (see Table 2). Annotated contigs from analyzed clusters are in supplementary files (Supplementary Files S8–10). Further details can be found in the RepeatExplorer summary and archives (Supplementary Files S11–13 and S14–16).

Unlike the comparative clustering, we detected not only previously observed long tandems but also several other satellites in separate clustering and TAREAN analyses. In *A. cepa*, we detected AcepSAT750 (CL62) and AcepSAT356 (CL15 and CL19) and, additionally, we detected AcepSAT2500 (CL147). In *A. sativum*, we detected AsatSAT189 (CL58) as previously but also AsatSAT604 (CL157), AsatSAT377 (CL120), and AsatSAT74 (CL143). In *A. ursinum*, the candidate AsatSAT89 was not detected in separate clustering. Compiled consensus sequences for all detected long tandem repeats (satellites and rDNA) can be seen in Supplementary File S1.

**Figure 7:**
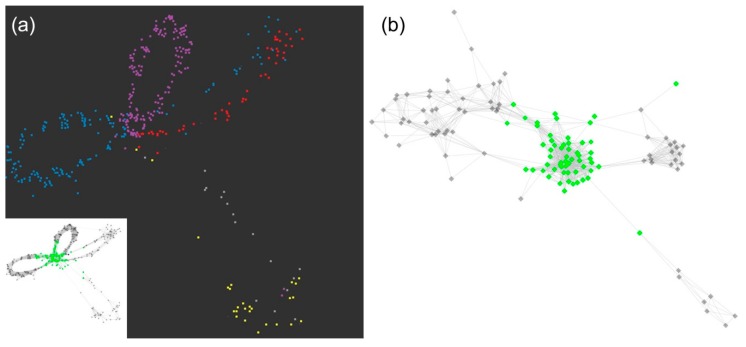
5S rDNA cluster in *A. sativum* and *A. cepa* in separate RepeatExplorer and TAREAN analyses. Dots represent reads, edges represent identified similarities (overlaps) between the reads. 5S rRNA gene reads are in green, intergenic spacers in black. (**a**) Cluster of 5S rDNA of *A. sativum*. The colours (dark graph) stand for specific contigs representing variability of the spacer (red—CL100Contig1, pink–CL100Contig2, grey—CL100Contig3 and CL100Contig4, blue—CL100Contig5, and yellow—CL100Contig5). (**b**) Cluster of 5S rDNA of *A.cepa*. The green core of the graph represents 5S rDNA genes and the two black loops represent two variants of the intergenic spacer in *A. cepa*.

We obtained interesting results in the separate clustering of 5S rDNA in *A. sativum* and *A. cepa*. There were reads covering the 5S rRNA gene in the core of the cluster, from which four and two distinct loops emanated, representing intergenic spacers in *A. sativum* and *A. cepa*, respectively. How six contigs from 5S rRNA genes and intergenic spacers from *A. sativum* (CL100Contig1–6; can be found Supplementary File S1) cover the cluster graph is depicted in Figure 7A. Three contigs for 5S rDNA in *A. cepa* (CL211Contig1–3; these can be found in Supplementary File S1) and the two variants of the intergenic spacer (loops in Figure 7B) were joined in the same contig (CL211Contig1). 

### 2.5. Fluorescence in Situ Hybridization (FISH)

#### 2.5.1. Telomere Sequence in A. sativum

**Figure 8:**
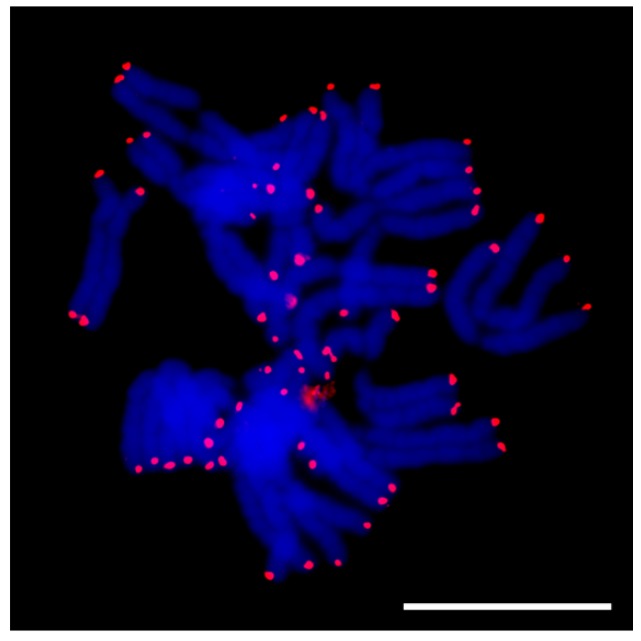
Fluorescence *in situ* hybridization of the telomere probe on *A. sativum* chromosomes. All termini show telomere signals and no interstitial localization can be seen. DAPI in blue, and specific probe in red. Scale bar, 10 µm.

Telomere sequences in *A. sativum* provide strong signals at all chromosomal termini. We did not detect any visible interstitial signals (Figure 8). *A. sativum* does not differ in telomere FISH signal from previously tested *A. cepa* and *A. ursinum* species [16].

#### 2.5.2. Short Tandem Repeats

**Figure 9:**
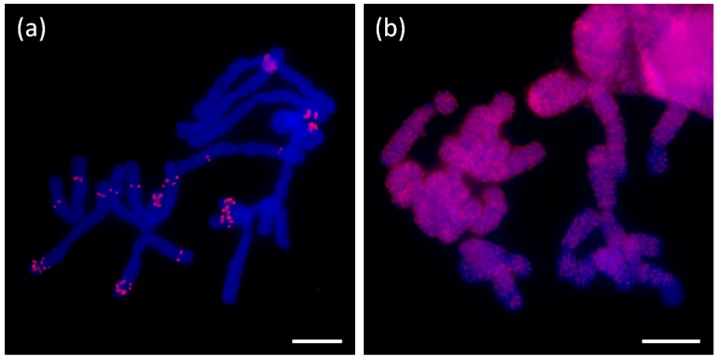
Fluorescence in situ hybridization with probes containing short tandem motifs. (**a**) Probe number 11 with the sequence of (TCGGATCGGT)*_n_* applied on chromosomes of *A. ursinum* (**b**) Probe number 5 with the sequence of (AATCGTTTTCAATTCAATTCATTTCGA)*_n_* on chromosomes of *A. sativum*. The specific probe signals are in red; DAPI staining in blue. The picture (**b**) exemplifies a dispersed signal pattern obtained for most of the STMs tested (Figure S6). Scale bar, 10 µm.

We tested 12 short tandem repeat motifs by Fluorescence *in situ* hybridization (FISH). The sequences selected and number of their detected reads are shown in Table 3. These repeats did not show chromosome specific localization in any species studied, except for probe number 11. This probe provided interstitial and also terminal signals with discrete localization on chromosomes of *A. ursinum*. In other cases, dispersed signals without any accumulation to specific areas were obtained, as demonstrated by the most abundant short tandem repeat in *A. sativum* (monomer length 27 nt; probe number 5; Figure 9). We did not obtain any signal with probes 9 and 10 (data not shown), and other FISH results with tested STM are shown in Supplementary Figure S6. The signal strengths mostly corresponded to the numbers of reads detected.

#### 2.5.3. Long Satellites

**Figure 10:**
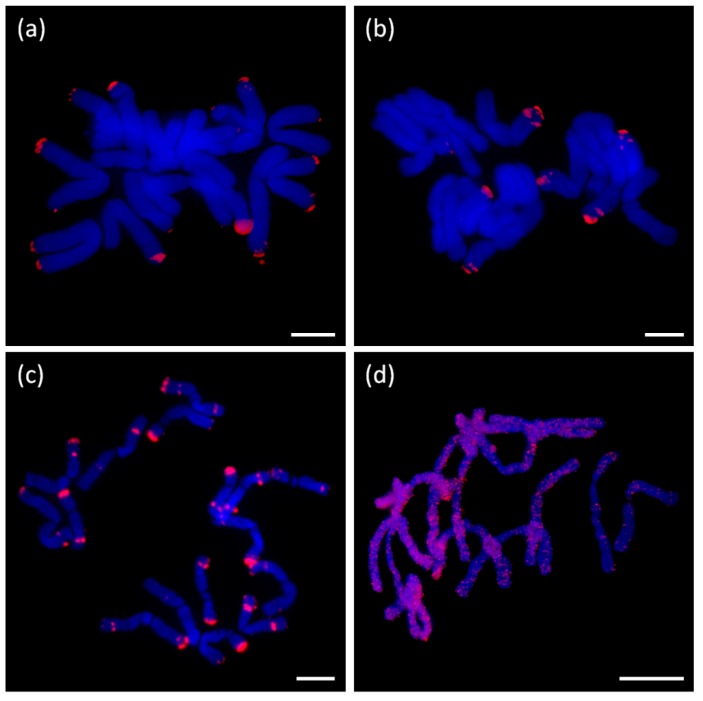
Fluorescence in situ hybridization using probes for long satellites and Ty1/copia/TAR (CL111 from comparative clustering). Localization of species-specific satellites: (**a**) detection of AcepSAT356 on chromosomes of *A. cepa*, (**b**) detection of AcepSAT750 on chromosomes of *A. cepa*, (**c**) detection of AsatSAT189 on chromosomes of *A. sativum*, and (**d**) detection of Ty1/copia/TAR on chromosomes of *A. ursinum*. Signals of specific probes are in red, and DAPI staining is in blue. Scale bar, 10 µm.

Probes corresponding to clusters annotated as long satellites (e.g., AcepSAT356) were localized to specific chromosomal loci. The signals of tested satellites were mostly sub-telomeric (AcepSAT356, AcepSAT750) on chromosomes of *A. cepa*. In the case of AsatSAT189, numerous sub-telomeric and interstitial signals on chromosomes of *A. sativum* were obtained (see Figure 10).

## 3. Discussion

We have performed a comprehensive pilot study of the repeatome in three *Allium* species, *A. cepa*, *A. ursinum* and *A. sativum*. All our results are based on incomplete and low genome coverage approaches (Table 4). Previously, similar analyzes were done in *A. fistulosum* [28,35] and *A. cepa* [31] but not in *A. sativum*, *A. ursinum* or any other species from the old *Allium* evolutionary line. Our selection of species for comparative analysis covered several aspects. *A. cepa* and *A. sativum*, two economically valuable crop species from the same young evolutionary line, are compared to each other and to the wild growing species from the old evolutionary line, *A. ursinum.* Our data are based on slightly higher genome coverage than in previous studies. For example, we used NGS data corresponding to 0.004× in comparative and 0.01× in separate TAREAN and RepeatExplorer analyses, and 0.277× in TRFi-TRM in the case of *A. cepa*. But it is not the coverage what makes the data specific. Considering their sensible volume, the data were produced in a way that minimized amplification bias (PCR-free libraries), and ensured at least minimal control of sequencing reliability (3× parallel libraries per species from the same DNA extract). 

We used two bioinformatic approaches. The first approach included Tandem Repeats Finder and the Tandem Repeats Merger tool to identify short tandem repeats. The second approach consisted of TAREAN and RepeatExplorer analyses. The estimated genomic proportion of repeats identified by all approaches together in all three species was roughly 63%. A list of identified short tandem repeats from TRFi-TRM is in the supplementary tables (Tables S1–4). In our estimation, these STMs represent 0.9–2.4% of the genome in the species studied. Repeats identified in RepeatExplorer represented ca. 60% of the genome in the three species (Figure 4). Clusters representing a genomic proportion of at least 0.01% were automatically analyzed in more detail. This analysis was focused on the similarity to already known repetitive elements using BLAST. Separate reads in clusters were assembled to contigs, and the clusters were depicted in graphs according to overlaps and information from pair-end mates. We were able to annotate most of them thanks to the automatic prediction in RepeatExplorer, TAREAN pipeline, and graphs shape (Tables S5–8). Only up to 4% of clusters analyzed in detail remain unannotated (Table 1 and Table 2). Nevertheless, a relatively large proportion of repeats remain unclassified (violet sector in Figure 6), most of these are clusters representing less than 0.01% of each genome. Very low- or single-copy sequences remained as non-clustered reads (grey sectors in Figure 6). Higher genomic coverage might uncover further repeats from this fraction as we hypothesize further in the discussion. The sequences from all, annotated and unannotated clusters can be found in Supplemetary Files S4 and S8–10. In total, the annotated fraction represents roughly 40–55% of the genomes studied (Table 1 and Table 2; Figure 6). 

Short tandem repeats with monomer lengths between three and 52 nucleotides, considering three adjacent monomers as the shortest arrangement, were analyzed in Tandem Repeats Finder and a set of custom made scripts called Tandem Repeat Merger [7,36]. In this way, we identified 136,968 tandem motifs in a sample of 40 million reads (10 million reads from *A. cepa* and *A. sativum* each, and 20 million reads from *A. ursinum*). We used a relatively high number of reads per species, yet the enormous sizes of the genomes studied means that even repeats detected in fewer reads represent a considerable DNA content. This fact is evident from recalculation demonstrated in Table 5. For example, numbers of reads with vertebrate-like and *Arabidopsis*-like telomere sequences in our TRFi-TRM result represent a genomic proportion corresponding to a short gene in the *Allium* genome context. Most of abundant tandem motifs in *A. cepa* belong to the category of genomic proportion corresponding to 160 kb–16 Mb, e.g., the telomere sequence corresponds to 406 kb in total in *A. cepa* (red bold frame) which also corresponds to the experimental results [16]. All detected motifs and estimations of their genomic proportions are shown in supplementary tables (Supplementary Tables S1–S4). 

An estimated total genomic proportion of short tandem repeats was 0.9% in *A. cepa*, 2.4% in *A. ursinum*, and 2.3% in *A. sativum*. However, the total number of identified motifs is probably overestimated as even the abundant motifs have rare mutant and degenerate variants that do not pass the TRFi-TRM threshold of tolerance for variability in the motif pattern to which they belong. There is also a strict limitation in the estimation of genomic proportion for tandems with monomer lengths approaching 50 nt. Such tandems have only three units in a read (151 nt), from which their consensus of the motif is calculated in TRFi. This fact itself probably decreases the success rate of tandem identification in the case of degenerate repeats. Moreover, the more degenerate the 50 nt tandem repeat is, the more incongruent are TRFi consensus sequences produced from distinct reads carrying this tandem. In the case of very short monomer lengths, e.g., 3 nt, the consensus produced was more representative and probably congruent for most reads with this tandem. Briefly, the shorter the motifs are, the more accurate the estimation of genomic proportion in terms of the number of motifs detected and their abundancy was. From this point of view, our results from degenerate tandems and tandems with motif lengths approaching 50 nt detected in TRFi-TRM are only semiquantitative and their value is mainly in qualitative information on their presence in the three *Allium* species and their comparison. In the case of conserved tandems such as telomere sequences, approaching the lower limit of motif lengths detected (minisatellites and microsatellites), the estimated genomic proportion is not burdened by the error of low motif representation in reads and their coverage corresponds to the abundancy. We estimate here that an average telomere length is 25.389 kb in *A. cepa*, 50.285 kb in *A. sativum*, and 90.998 kb in *A. ursinum*. Although the genome proportion of telomere sequence in tested species was very low (0.002–0.005%), the estimated average telomere length in several tens of kilobase pairs ranks *Allium* among plants with rather long telomeres. For example, the longest terminal fragments in plants were detected in *Nicotiana tabacum* (160 kb) [37] and *Nicotiana sylvestris* (200 kb) [38] while most ecotypes of *Arabidopsis thaliana* range between 3.5–9 kb [39,40].

Interestingly, we also detected vertebrate-like and *Arabidopsis*-like telomere sequences in genomic DNA of all three species. The detected numbers of reads mostly represent a very small DNA content that could be a trace of an ancient telomere sequence in the *Allium* ancestor (the number of *Arabidopsis*-like reads in library S2, 1843 (Table 3), was neglected as an outlier or experimental artifact). This actually exemplifies the reason why three parallel libraries were used for each species. If the other two libraries were not prepared (the number of *Arabidopsis*-like reads in library S1–8 reads and S3–6 reads (Table 3)), we would not be able to recognize the value of this repeat of interest in S2 library as irrelevant. In the case of the vertebrate-like sequence in *A. sativum* and the *Arabidopsis*-like sequence in *A. ursinum*, the number of reads detected corresponds to several kb (Table 5). This finding corresponds to the previously published occurrence of both repeats at non-terminal positions in *Allium* species [32]. Other short tandem repeats selected for FISH localization did not provide any specific signal. In all cases, the FISH signal was interspersed throughout the genome with diverse intensity, which was congruent with the number of reads detected per tandem. The only specific terminal localization was detected in the case of telomere short tandem repeats (CTCGGTATTGGG)*_n_* in *A. sativum*, *A. cepa*, and *A. ursinum* (Figure 8 and [16]) and (TCGGATCGGT)*_n_* in *A. ursinum* (at interstitial and terminal loci; see Figure 9a). Neither interstitial nor terminal signals were detected in *Arabidopsis*- or vertebrate-like telomere sequences.

Our results with slightly higher content of TTTAGGG than TTAGGG in *A. cepa* and *A. ursinum*, and comparable numbers of both in *A. sativum* raise the question of ancestral telomere sequences in the *Allium* genus. It is not clear whether the ancestor’s telomerase synthesized purely (TTAGGG)*_n_* or a mixed array of (TTAGGG/TTTAGGG)*_n_* as was shown for some Asparagales [33]. 

Major repeats, particularly mobile elements, rDNA and satellites, were characterized in the RepeatExplorer analysis (Supplementary Tables S5–8 and Files S8–10). DNA transposons (mobile elements class II) represented only a tiny genome proportion (up to 0.07%). It is consistent with the “cut and paste” mode of mobilization which does not result in an expansion comparable to retrotransposons (mobile element class I). Generally, we conclude that the main contribution to all three genomes is by an abundancy of the Ty3/gypsy element Tekay (ca. 30% in *A. cepa*, 45% in *A. ursinum*, and almost 50% in *A. sativum*). It is not clear whether the whole captured genomic proportion of such large repeats like retrotransposons correspond to fully functional variants. It is probable that incomplete, truncated and other derived variants were designated with the annotation of the full repeat, which may represent only a part of the specific cluster or supercluster. Only if the short version formed a very specific and considerably abundant population of copies in the genome, we would be able to annotate it as a separate element, such as in the case of TRIMs (Table 1 and Table 2). Nevertheless, a vast majority of detected retrotransposons, including TRIM elements, were identified in species-specific clusters regardless of their classification. Similar situation has already been studied in the comparative analysis of several *Fritillaria* (Liliaceae) species with giant genomes. The study indicated, that the genome expansion occurred independently via accumulation of variable repeats with the lack of DNA removal [41]. The repeatome profiles of obese *Fritillaria* genomes were shown to be dramatically different, particularly in the presence of the species-specific satellites [42]. A different observation was made in the case of *Allium* in rather rare but conserved Ty1/copia/TAR element (CL111 in the Supplementary File S2, Figure S3 shows mapping of contigs from CL111 to annotated hypothetical copy (5417 nt) as a reference sequence). Conservation of this repeat gave an impression that it could be connected to some specific loci, like centromeres or subtelomeres. Unexpectedly, using FISH, we did not find any enrichment of this element in any specific locus (Figure 10d). 

Surprisingly, the genomic proportion of detected repeats is more similar in *A. sativum* and *A. ursinum* than between more closely related *A. cepa* and *A. sativum* (Figure 6). The representativeness of the profiles should be guaranteed by technical triplication of NGS libraries from each species and PCR-free library preparation.

Contrary to most retrotransposons and long satellites, rDNA sequences from all three species were clustered in the same group, as expected according to their conserved character. We made several interesting observations in these rDNA clusters. In the case of 5S rDNA, a complete circular cluster shape was detected in *A. cepa* and *A. sativum*. In *A. ursinum*, the part corresponding to a spacer between 5S rRNA genes was not completely assembled. It is possible that the variability of its sequence makes it impossible to assemble the spacer variants under the coverage used in RepeatExplorer. Remarkably, more reads from 5S rDNA were detected in *A. sativum* (Figure 5). The shape of clusters indicates the presence of four variants in *A. sativum* and two intergenic spacer variants in *A. cepa* (Figure 7). Notably, *A. sativum* has additional 5S loci compared to *A. cepa* [19,20]. Further analysis is needed to localize these variants and to show whether they occupy the same or distinct chromosomal loci. In 45S rDNA clusters, *A. sativum* exhibited a single circular cluster of the long tandem array (Supplementary Figure S4a). *A. cepa* 45S rDNA was assembled into two clusters that form one circular supercluster through mate pair reads. Therefore, they have been merged to obtain consensus and its variants (Figure S4c,d; File S17). Interestingly, the assembled 45S rDNA from *A. cepa* cultivar Všetana is more similar to a partial clone from *A. cepa* cultivar Ailsa Craig (GenBank EU256494.1), [43] than to the complete onion 45S rDNA sequence published recently (GenBank KM117265), (Figure S5). Unexpectedly, in *A. ursinum*, the graph of the 45S rDNA cluster was not enclosed in the circular shape, which is typical for tandem arrangement and stayed linear, Figure S4B. It happened probably due to the presence of a long array of GC rich short tandem repeats in its spacer region, which can be a difficult substrate for enzymes used in NGS. 

We identified new satellite sequences in *A. cepa*, *A. ursinum* and *A. sativum*. We named these according to previous practice (similar to ACSAT from [25]) according to species and monomer length. Since we expect more NGS data from other *Allium* species to be published soon we suggest using a longer prefix, such as Acep for *A. cepa*, not to be confused, e.g., with *A. cernuum*. Further modification and unification of nomenclature will be probably needed once the other species will be analyzed (e.g., *A. cepiforme*). The newly identified satellites are AcepSAT750 and AcepSAT2500 from *A. cepa*, and AsatSAT377, AsatSAT 189, AsatSAT 74, and AsatSAT 604 from *A. sativum*. A putative satellite was detected in *A. ursinum* and was termed AursSAT89. AcepSAT750, AcepSAT356, and AsatSAT189 satellites have specific chromosomal locations and potentially, these can be used as chromosomal markers. In comparison to similar previously described satellites from *A. cepa*, e.g., pAc074 and ACSAT, which are 314–378 nt long [23,24,25,27,30], we identified a consensus with a length of 356 nt and termed it AcepSAT356. This consensus, which actually stands for one major variant, was examined by FISH. We obtained a terminally positioned signal. However we observed a lack of signal at more than just one chromosomal arm (Figure 10a). The satellite was previously estimated to constitute up to 4% of the genome in *A. cepa* [23]. Our NGS results show the proportion to be 0.28% in a comparative analysis and 1.3% in separate clustering with higher genomic coverage. This difference can be interpreted either as a low precision of the previous analyses, or as a result of a low genomic coverage in our analyses and, consequently, its biased quantitative result (Table 4). Increased genomic coverage would probably minimize the bias but the input used is at the limit of the server with the web based pipeline of RepeatExplorer and TAREAN.

Some *Allium* retrotransposon lineages such as Ty1/copia Bianca, Ikeros, Ale, and Angela are currently near the limit of detection by RepeatExplorer. They were not detected using a genomic coverage of 0.004× and only when the genomic coverage was increased (0.007–0.01×) did they become obvious in the dataset as well as in the DNA element MITE from *A. cepa*. It is possible that by further extending the amount of sequences analyzed, we will detect other lineages of repeats with tiny genomic proportions. This hidden variability may uncover a substantial part of unclassified and non-clustered sequences in our study, mainly those of low copy number and high sequence variability. 

*A. cepa*, *A. ursinum*, and *A. sativum*, with our estimations (based on the result from FastQC output [44] on raw whole genome shotgun sequencing data) of 33% GC content, were shown to be one of the AT- richest plant species [45,46]. On the other hand, experimental measurement (39% GC) shows *Allium* as rather an average plant genome rather than any extreme, with a low GC content [47]. In this light, our results rather reject the hypothesis that the low GC content in *Allium* is caused by expansion of non-genic tandem repeats. Although short AT rich tandem motifs were much more frequent than GC rich ones, STM altogether constituted only a few percent of the genome in the species studied (up to 0.9% in *A. cepa*). Long satellites were estimated to constitute slightly, yet not dramatically, larger genomic proportions (1.6–1.7% in *A. cepa*). Moreover, the satellites differed in GC content and were rarely below 30%. In contrast, *A. cepa* retrotransposons, e.g., Ty1/copia/TAR were richer in AT than the most prevalent long satellite, AcepSAT356 (Table 6). 

## 4. Materials and Methods

### 4.1. Plant Species

We used three species from the genus *Allium* (Amaryllidaceae) in this study: *Allium cepa* cv. Všetana (seeds from SEVA-FLORA S.R.O.; Valtice, Czech Republic, www.sevaflora.com), *A. sativum* cv. Havran (bulbs from Ing. Vávlav Kozák; Holice, Czech Republic, www.cesnek.cz), and *A. ursinum* (plant material collected in Černovice, Czech Republic). *A. ursinum* belongs to the oldest evolutionary line, *A. cepa* and *A. sativum* are rather distant members of the youngest evolutionary line. The distance between *A. cepa* and *A. ursinum* is probably larger than between *A. sativum* and *A. ursinum*, however, the relations inside lineages are not entirely clear [1]. For the genome size conversion from pg to bp, we used the recalculation that 1 pg of DNA corresponds to ca. 978 Mbp [48]. This means that *A. cepa* has 16.38 Gb, *A. sativum* 15.89 Gb, and *A. ursinum* 30.76 Gb per haploid genome. All species used in this study are diploids.

### 4.2. DNA Extraction for NGS

We collected 1g of either seedlings (*A. cepa*) or young leaves (*A. sativum* and *A. ursinum*) and carried out DNA extraction in a conventional way as described in [49], including a phenol:chloroform purification step. 

### 4.3. Next-Generation Sequencing

We prepared three DNA aliquots (10 µL; ~1 µg/µL) of each sample in parallel (samples 1–9; S1–S3 from *A. cepa*; S4–S6 from *A. ursinum*; and S7–S9 from *A. sativum*). These nine samples were sent to the NGS service provider (Admera Health, LLC, South Plainfield, NJ, USA). We requested a preparation of nine PCR-Free libraries using KAPA High Throughput Library Preparation Kit with SPRI solution and no PCR Library Amplification/Illumina series (Kapa biosystems a Roche Company, Wilmington, DE, USA). An average size of genomic inserts was 500 bp. Multiplexing of libraries and 151 nt long pair-end reads were ordered.

### 4.4. NGS Data Preprocessing

Quality control was performed in FastQC [44] and further preprocessing was done as described in [7,50]. Read headers from each library containing unique paired-end information were provided with prefixes (S1–S9) and suffixes (1/2) according their sample-library belonging and mate information. 

### 4.5. TRFi and TRM

Short tandem motifs were analyzed in unassembled NGS reads regardless of their pair mate information. We analyzed 10 million reads from *A. cepa* and *A. sativum* each and 20 million reads from *A. ursinum* in TRFi/TRM with the default setting of TRFi for NGS data. Exceptionally for the MaxPeriod option, we extended it up to 55. TRM used for the merging of detected identical tandem motifs (regardless of their orientation or rotation, e.g., TTA, TAT, ATT, AAT, ATA, and TAA) and counting of corresponding reads was set to analyze motifs with a length of at least three nucleotides, repeated at least three times in adjacent arrays. For more details see [7]. 

### 4.6. RepeatExplorer and TAREAN

We performed comparative analysis in RepeatExplorer and TAREAN with an input of 1,800,000 PE reads in total (Supplementary File S18). There are 450,000 reads from *A. cepa* and *A. sativum* (each) and 900,000 reads from *A. ursinum*. Inputs for separate analyzes were of 1,800,000 PE reads each, however the number of analyzed sequences was always lower due to automatic downsizing of the input (Supplementary File S19–21). The raw NGS data were published in BioProject (GenBank: PRJNA512235; SRR8380541-9). The analysis depth of these efficient tools is determined by several factors, such as genome size, complexity, and the number of available reads. The *Allium* genomes are rather giant (tens of Gb per haploid genome) and the current resource allocation (up to 16 CPUs, 1792 Gb RAM) to RepeatExplorer Galaxy server [51] effectively processes inputs of around 0.8–1.8 million reads per *Allium* genome in one clustering run. The data always represent random samples of all independent PCR-free libraries for comparative analyses or three species libraries for separate analyzes. The setup of both tools was the default with a true option of paired-end reads and comparative analysis (group code length = 2).

### 4.7. Annotation

Annotation was done in two steps. The first step is an automatic annotation and prediction as a component of RepeatExplorer and TAREAN workflow. This prediction was manually verified and edited. The final annotation was done at the level of contigs from superclusters (clusters connected via paired-end read mates). For example, all contigs from clusters that belong to a supercluster in which RepeatExplorer identified conserved domains of Ty3/gypsy Tekay group in typical order were annotated as this repeat. However, some clusters represent variable regions of specific sub-lineages etc. In this way, we annotated contigs from comparative analysis (supplementary File S4) and separate analyzes, respectively (Supplementary Files S8–10). The information about automatic and manual annotation and prediction as well as about estimations of genomic proportions can be found in the analysis archives (Supplementary Files S3 and S14–16) and processing tables (Supplementary Tables S5–8). Annotation of major repeats is shown directly in the supplementary files with identified sequences (Supplementary Files S4 and S8–10). There are also separate files with sequences of plastid DNA (Supplementary Files S5–7) and tandem repeats (Supplementary Tables S1–4) available in the supporting information.

### 4.8. Chromosomal Preparations

Chromosome spreads from root tips were prepared according to the published protocol [52]. Actively growing, young roots were harvested from seedlings, cultivated, and collected plants, pre-treated with ice-cold water for 12 h, fixed in ethanol/acetic acid (3:1, *v*/*v*) fixative for 24 h at 4 °C and stored at −20 °C until further use. Selected root tips were rinsed in distilled water (twice for 5 min) and citrate buffer (10 mM sodium citrate, pH 4.8; twice for 5 min), and digested in 0.3% (*w*/*v*) cellulase, cytohelicase and pectolyase (all Sigma-Aldrich) in citrate buffer at 37 °C for 90 min. After digestion, individual root tips were dissected on a microscope slide in approximately 10 µL of acetic acid and covered with a cover slip. The cell material was then spread evenly using tapping, thumb pressing, and gentle flame-heating. Finally, the slide was quick frozen in liquid nitrogen and the cover slip flicked off with a razor blade. Slides were fixed in ethanol/acetic acid (3:1) and air-dried. Chromosomes were counterstained with 2 µg/mL DAPI in Vectashield (Vector Laboratories).

### 4.9. FISH Probes

Oligonucleotide probes were designed from consensus DNA sequences of short tandem repeat sequences (Table 3), long satellites (AcepSAT356, AcepSAT750, AsatSAT189, and AursSAT89) and Ty1/copia/TAR element (CL111). Target sequences were manually selected to obtain as high level of sequence complexity as possible to maximize probe specificity and GC content. The double-stranded DNA probes were generated from oligonucleotides by mixing complementary ones, heating up to 95 °C and gradual cooling to room temperature in a water bath. The probes were labelled with biotin-dUTP, digoxigenin-dUTP, or Cy3-dUTP by nick translation as described by Mandáková and Lysak [53]. The set of oligonucleotides used is shown in the supplementary file Table S9.

### 4.10. Fluorescence in Situ Hybridization and Microscopy

Labelled probes were pooled to follow the design of a given experiment, ethanol precipitated, dried and dissolved in 20 μL of 50% formamide and 10% dextran sulfate in 2× SSC per slide. The 20 μL of the labelled probe were pipetted on a chromosome containing slide and immediately denatured on a hot plate at 80 °C for 2 min. Hybridization was carried out in a moist chamber at 37 °C overnight. Post-hybridization washing was performed in 20% formamide in 2× SSC at 42 °C. The immunodetection of hapten-labelled probes was performed as described by Mandáková and Lysak [53] as follows: biotin-dUTP was detected by avidin–Texas Red (Vector Laboratories, Burlingame, CA, USA) and amplified by goat anti-avidin–biotin (Vector Laboratories) and avidin–Texas Red; digoxigenin-dUTP was detected by mouse antidigoxigenin (Jackson Immuno Research, Cambridgeshire, United Kingdom) and goat anti-mouse–Alexa Fluor 488 (Invitrogen, Waltham, MA, USA). Chromosomes were counterstained with 2 μg/mL DAPI in Vectashield. The preparations were photographed using a Zeiss Axioimager Z2 epifluorescence microscope with a CoolCube camera (MetaSystems, Altlussheim, Germany). Images were acquired separately for all four fluorochromes using appropriate excitation and emission filters (AHF Analysentechnik, Tübingen, Germany). The four monochromatic images were pseudocolored, merged, and cropped using Photoshop CS (Adobe Systems, San Jose, CA, USA).

## 5. Conclusions

In this work we describe major repetitive elements from nuclear genomes of three *Allium* species. Identified sequences of repeats are annotated and available in FASTA format file. We also created a profile of short tandem repeats for each of the species studied and assembled long contigs of plastid DNA de novo. 

The data presented here can be used in a number of ways, e.g., as a reference for a search of centromere repeats in *A. cepa*, *A. ursinum* and *A. sativum* in which the centromere repeat has not been identified so far. Retrotransposons from the Ty3/gypsy group are enriched in centromeric heterochromatin and, thus, are suitable candidates to examine their centromeric functions using a Chip-Seq approach. Further, our repeat databases provide a source to mine chromosome specific markers, as we showed in the case of long satellites in this work, or as was previously shown in *A. fistulosum* with CAT36 and HAT58 [28]. Identified markers are potentially usable in breeding approaches including interspecific hybrids.

The genome sampling in the NGS skimming approach used here with RepeatExplorer may credibly reflect the accuracy and completeness of genome assembly de novo. Still, this is the first attempt at clustering PCR-free data in *A. cepa* and two other species, which was limited by the RepeatExplorer server capacity. More computational resources are needed to uncover so far unassembled and unclassified repeats from the violet and most of the grey sectors in the pie graph (Figure 6) using the RepeatExplorer pipeline. 

For long tandem repeats and long arrays of tandem repeats, such as 45S rDNA or telomeres, the RepeatExplorer abundancy estimation presented in this study might be more accurate than the analysis of the assembled genome itself. The complete assembly of long tandem arrays as they would reflect some real block of DNA is still a challenging task which is biased particularly in repeats with high conservativeness and low complexity. The approach of clustering in RepeatExplorer, TAREAN, and sampling in TRFi-TRM, without ambition to assemble longer tracts than a repeat unit, result in a straightforward unit identification de novo and determination of its frequency in the genome. Potentially, our species-specific repeatome database can be used for efficient annotation or masking loci containing repeats in assembled corresponding genomes because it contains information not only about conservative domains of repeats but also about variability in less-conserved parts of repeats.

## Figures and Tables

**Table 1:** Genome proportion of annotated repeats in all three species in a comparative analysis.

Repeat Annotation	Genomic Proportion %		
	*A. cepa*	*A. ursinum*	*A. sativum*
mobile_element/Class_II/Subclass_1/TIR/EnSpm_CACTA	0.12	0.64	0.07
mobile_element/Class_I/LINE	9.18 × 10^−4^	0.27	6.90 × 10^−4^
mobile_element/Class_I/SINE	4.59 × 10^−4^	0.17	0.09
mobile_element/Class_I/LTR/Ty1_copia/SIRE	3.12	1.11	0.58
mobile_element/Class_I/LTR/Ty1_copia/TAR	0.28	0.44	0.29
mobile_element/Class_I/LTR/Ty1_copia/Tork	0.00	0.05	0.00
mobile_element/Class_I/LTR/Ty3_gypsy/chromovirus/CRM	0.66	1.96 × 10^−3^	0.00
mobile_element/Class_I/LTR/Ty3_gypsy/chromovirus/Tekay	24.86	45.51	47.88
mobile_element/Class_I/LTR/Ty3_gypsy/non-chromovirus/OTA/Athila	0.08	0.14	0.13
mobile_element/Class_I/LTR/Ty3_gypsy/non-chromovirus/OTA/Ogre_Tat/TatV	3.22	4.30	1.16
rDNA/45S_rDNA	0.38	0.26	0.33
rDNA/5S_rDNA	0.01	2.08 × 10^−3^	0.05
satellite	1.68	0.17	0.31
mobile_element/Class_I/LTR/TRIM	0.13	0.16	0.53
unknown in analyzed clusters	2.12	0.73	1.26
**total in analyzed clusters**	**36.65**	**53.95**	**52.68**
small clusters that were not analyzed	16.74	11.92	11.43
non-clustered reads	46.61	34.13	35.89
total	100.00	100.00	100.00

**Table 2:** Genomic proportion of annotated repeats in all three species in separate clustering. Comparison of repeat elements according to their belonging to repeat class and lineage.

Repeat Annotation	Genome proportion %		
	*A. cepa*	*A. ursinum*	*A. sativum*
mobile_element/Class_II/Subclass_1/TIR/EnSpm_CACTA	0.02	0.73	0.00
mobile_element/Class_II/Subclass_1/TIR/MITE	0.03	0.00	0.00
mobile_element/Class_I/LINE	0.00	0.36	0.03
mobile_element/Class_I/SINE	0.06	0.13	0.14
mobile_element/Class_I/LTR/Ty1_copia/SIRE	3.97	1.13	0.30
mobile_element/Class_I/LTR/Ty1_copia/TAR	0.27	0.51	0.35
mobile_element/Class_I/LTR/Ty1_copia/Tork	0.05	0.04	0.12
mobile_element/Class_I/LTR/Ty3_gypsy/chromovirus/CRM	0.73	0.00	0.00
mobile_element/Class_I/LTR/Ty3_gypsy/non-chromovirus/OTA/Ogre_Tat	0	8.71 × 10^−3^	0
mobile_element/Class_I/LTR/Ty3_gypsy/non-chromovirus/OTA/Ogre_Tat/TatV	4.73	4.29	1.41
mobile_element/Class_I/LTR/Ty3_gypsy/chromovirus/Tekay	29.67	44.46	49.97
mobile_element/Class_I/LTR/Ty3_gypsy/chromovirus	0.00	2.85	0.00
mobile_element/Class_I/LTR/Ty3_gypsy/non-chromovirus/OTA/Athila	0.13	0.28	0.64
mobile_element/Class_I/LTR/TRIM	0.00	0.18	0.52
mobile_element/Class_I	0.00	5.64 × 10^−3^	0.00
mobile_element/Class_I/LTR/Ty1_copia/Bianca	0.02	0.01	0.05
mobile_element/Class_I/LTR/Ty1_copia/Ikeros	0.01	0.00	0.00
mobile_element/Class_I/LTR/Ty1_copia/Ale	0.00	0.00	0.01
mobile_element/Class_I/LTR/Ty1_copia/Angela	0.00	6.08 × 10^−3^	0.11
rDNA/45S_rDNA	0.37	0.26	0.31
rDNA/5S_rDNA	0.01	5.27 × 10^−3^	0.06
satellite	1.64	0.12	0.36
unknown in analyzed clusters	3.93	2.99	1.48
**Total in analyzed clusters**	**45.66**	**58.36**	**55.84**
unknown small clusters that were not analyzed	14.45	10.60	11.05
non-clustered reads (low/single)	39.89	31.04	33.11
total	100.00	100.00	100.00

**Table 3:** List of short tandem motifs selected for FISH and their read number per species.

Probe	Tandem Repeat Unit	Number of Detected Reads								
		*A. cepa*			*A. ursinum*			*A. sativum*		
		S1	S2	S3	S4	S5	S6	S7	S8	S9
1	TTAA	4636	4481	4651	1207	1251	1265	135	100	124
2	TATG	4279	4088	4349	589	646	616	1086	984	1055
3	TTTAAAATAG	646	589	671	4	4	2	2	3	2
4	TTTGTGCCTTCGGG	321	294	340	0	0	3	0	1	1
5	AATCGTTTTCAATTCAATTCATTTCGA	31	94	16	56	74	58	10748	10846	10322
6	ATGAATATCAATTCAACAC	11	19	13	19	21	26	4369	4471	4237
7	TTGAA	48	61	34	762	775	776	3737	3831	3598
8	AAATAGAAATATTTG	23	36	7	21	19	20	3793	3838	3803
9	TTAGGG	1	4	4	1	2	1	12	20	18
10	AAATATATTTGGGAT	4	2	2	2541	2575	2514	5	5	3
11	TCGGATCGGT	1	8	2	2297	2254	2177	2	5	4
12	TTTAGGG	8	1843	6	23	14	24	6	14	11

**1**—the most abundant short tandem motif (STM) in *A. cepa* and obviously less represented in the other species. **2**—the most abundant tetranucleotide tandem in *A. sativum*, however an even higher amount of it was detected in *A. cepa*. **3**—the most abundant 10-nucleotide tandem in *A. cepa*. **4**—a species specific tandem motif in *A. cepa*. **5**—a tandem 27-nucleotide motif representing the largest genomic proportion if we consider all STM from all three species, but it is rather species-specific for *A. sativum*. **6**—the most abundant 19-nucleotide tandem in *A. sativum*, **7**—the most abundant pentanucleotide motif in *A. sativum*. **8**—the most frequent 15-nucleotide motif in *A. sativum*. **9**—interestingly, some reads containing vertebrate-like telomere sequences are present in all species but slightly more in *A. sativum*. **10** and **11** represent the most frequent motifs according to their motif lengths. **12**—some reads containing *Arabidopsis*-like telomere sequences are present in all species but slightly more in *A.ursinum* (outlier value, 1843, in the *A. cepa* S2 is probably an artefact of NGS processing).

**Table 4:** Genome coverage used in comparative or separate clustering, and TRFi-TRM analyzes.

Species	Genome Size (bp)	Comparative Analysis		Separate Analysis		TRFi-TRM	
		No. of Analyzed Reads Without Plastid Sequences	Genome Coverage	No. of Analyzed Reads Without Plastid Sequences	Genome Coverage	No. of Analyzed Reads	Genome Coverage
*A. cepa*	1.64 × 10^10^	435,527	0.004	1,129,077	0.010	30,000,000	0.277
*A. ursinum*	3.08 × 10^10^	866,802	0.004	1,365,659	0.007	60,000,000	0.295
*A. sativum*	1.59 × 10^10^	434,945	0.004	754,189	0.007	30,000,000	0.285

**Table 5:** Recalculation of reads with short tandem motifs in 10 million reads per sample in *A. cepa* and *A. sativum* and 20 million reads per sample in *A. ursinum.* The left part of this table is the calculation to a hypothetical genomic proportion, a relative, and absolute DNA content. The right part compares categories of well-known objects.

Species	nr. Reads Detected	Relative Frequency	% Genome Proportion	bp / 1 Genome	Genomic Element with Comparable Proportion
*A. cepa* & *A. sativum* (16 Gb)	1	0.0000001	0.00001	1600	Short gene
	10	0.000001	0.0001	16,000	~1/4 telomere loci *A. thaliana* (2n; Columbia)
	100	0.00001	0.001	160,000	distinct abundant motifs in *Allium* (e.g., telomere CTCGGTTATGGG represents 406 kb/haploid genome *A. cepa*)
	1000	0.0001	0.01	1,600,000	
	10,000	0.001	0.1	16,000,000	
	100,000	0.01	1	160,000,000	hapl. genome *A. thaliana* (all motifs in *A. cepa*)
	1,000,000	0.1	10	1,600,000,000	1/2 hapl. human genome
	10,000,000	1	100	16,000,000,000	hapl. genome *A. cepa*
*A. ursinum* (32 Gb)	1	0.00000005	0.000005	1600	Short gene
	10	0.0000005	0.00005	16,000	~1/4 telomere loci *A. thaliana* (2n; Columbia)
	100	0.000005	0.0005	160,000	distinct abundant motifs in *Allium* (e.g.,
	1000	0.00005	0.005	1,600,000	telomere CTCGGTTATGGG represents 406
	10,000	0.0005	0.05	16,000,000	kb/haploid genome *A. cepa*)
	100,000	0.005	0.5	160,000,000	hapl. genome *A. thaliana* (all motifs in *A. cepa*)
	1,000,000	0.05	5	1,600,000,000	1/2 hapl. human genome
	10,000,000	0.5	50	16,000,000,000	hapl. genome *A. cepa*

**Table 6:** Comparison of GC/AT content in the *A. cepa* repeats analyzed in TAREAN. Notably, most of long satellites are more than 40% GC rich.

Satellite	Monomer Length	A/T	G/C	GC%	AT%
AcepSAT750	750	454	296	39.5	60.5
AcepSAT356	356	188	168	47.2	52.8
AcepSat356	356	190	166	46.6	53.4
AcepSAT2500	2500	1804	696	27.8	72.2
45S rDNA	8614	4332	4282	49.7	50.3
5S rDNA	342	196	146	42.7	57.3
Ty1/copia/TAR CL111	5417	3364	2053	37.9	62.1

## Supplementary Materials

Supplementary materials (Figure S1–6; File S1, File S4–10, and Table S1–9) can be found at http://www.mdpi.com/1422-0067/20/3/733/s1. Due to an extensive volume of supplementary data (File S2, S3, and S11–21), complete supplementary files are available online at webpage: https://www.ibp.cz/local/data/allium/.

## Abbreviations

FASTA	Text-based format for representing sequences with their names, comments and annotation (abbreviation of fast all)
HTML	Standard markup language for creating web pages and web applications (Hypertext Markup Language)
AALSAT	*Allium altaicum* satellite
AcepSAT	*Allium cepa* satellite
ACSAT	*Allium cepa* satellite
AFISAT	*Allium fistulosum* satellite
AGASAT	*Allium galanthum* satellite
AOSAT	*Allium oschaninii* satellite
AROSAT	*Allium roylei* satellite
AsatSAT	*Allium sativum* satellite
AursSAT	*Allium ursinum* satellite
bp	Base pairs
CAT36	periCentromeric *Allium* tandem repeat from CL36
CL	Cluster
CPU	Central processing unit
DAPI	4′,6-Diamidino-2-phenylindole
FISH	Fluorescence in situ hybridization
Gb	Giga base pairs
HAT58	Heterochromatin-associated *Allium* tandem repeat from CL58
LINE	Long interspersed nuclear element
Mbp	Mega base pairs
NGS	Next generation sequencing
nt	Nucleotide
PCR	Polymerase chain reaction
RAM	Random-access memory
rDNA	Ribosomal DNA, DNA with ribosomal RNA genes
rRNA	Ribosomal RNA, RNA involved in structure of ribosomes and proteosynthesis
S1-S9	Samples 1–9, headers of reads in FASTA format were provided with prefixes according their belonging
SC	Supercluster
SINE	Short interspersed nuclear element
STM	Short tandem motif
TAREAN	Tandem repeat analyzer
TRFi-TRM	Tandem repeats finder—Tandem repeats merger
TRIM	Terminal-repeat retrotransposons in miniature
