# Supplementary material for: Comparative Dissection of Three Giant Genomes: Allium cepa, Allium sativum, and Allium ursinum
Source: Int J Mol Sci. 2019 Feb 9;20(3):733. doi: 10.3390/ijms20030733 (PMC6387171; doi:10.3390/ijms20030733)
Supplement: Supplementary file 1 [file ijms-20-00733-s001.zip › 5.ijms-430914-S/suppl_figure/Figure_S2.docx]

**
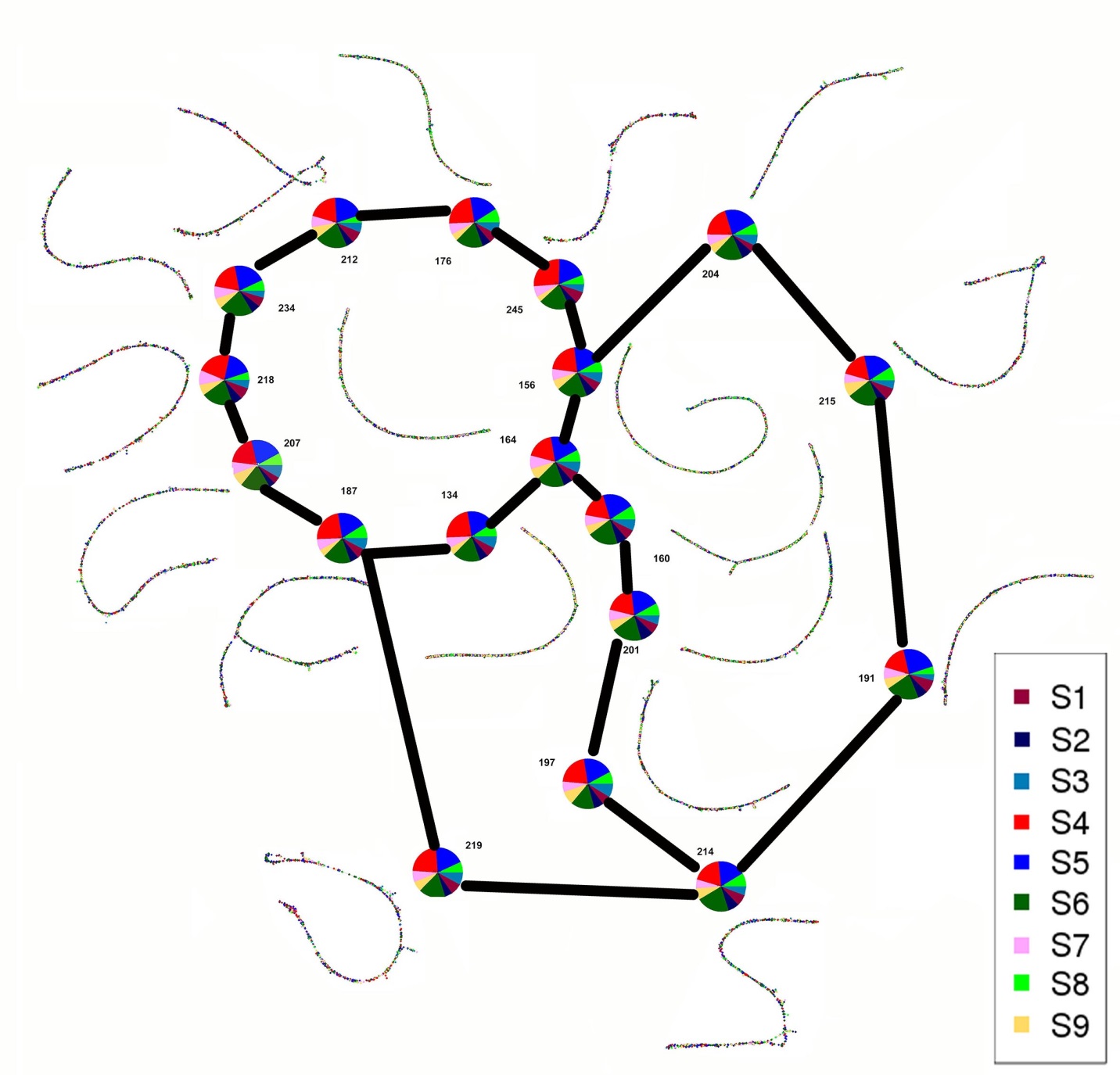
**

**Figure S2.** Plastid supercluster number 40 from comparative RepeatExplorer analysis. Based on the mate pairs information, we manually joined separate plastid clusters (color discs) with linear shaped graphs (miniature of the graph near each disc) and reconstructed supercluster of plastid DNA in studied species. The clusters belonging to samples is represented by colors (S1–3: *A. cepa*, S4–6: *A. ursinum*, S7–9: *A. sativum*). The plastid supercluster has circular shape and the clusters have very thin contours which together represents high level of homology in all three species.
