# Supplementary material for: Comparative Dissection of Three Giant Genomes: Allium cepa, Allium sativum, and Allium ursinum
Source: Int J Mol Sci. 2019 Feb 9;20(3):733. doi: 10.3390/ijms20030733 (PMC6387171; doi:10.3390/ijms20030733)
Supplement: Supplementary file 1 [file ijms-20-00733-s001.zip › 5.ijms-430914-S/suppl_figure/Figure_S3.docx]

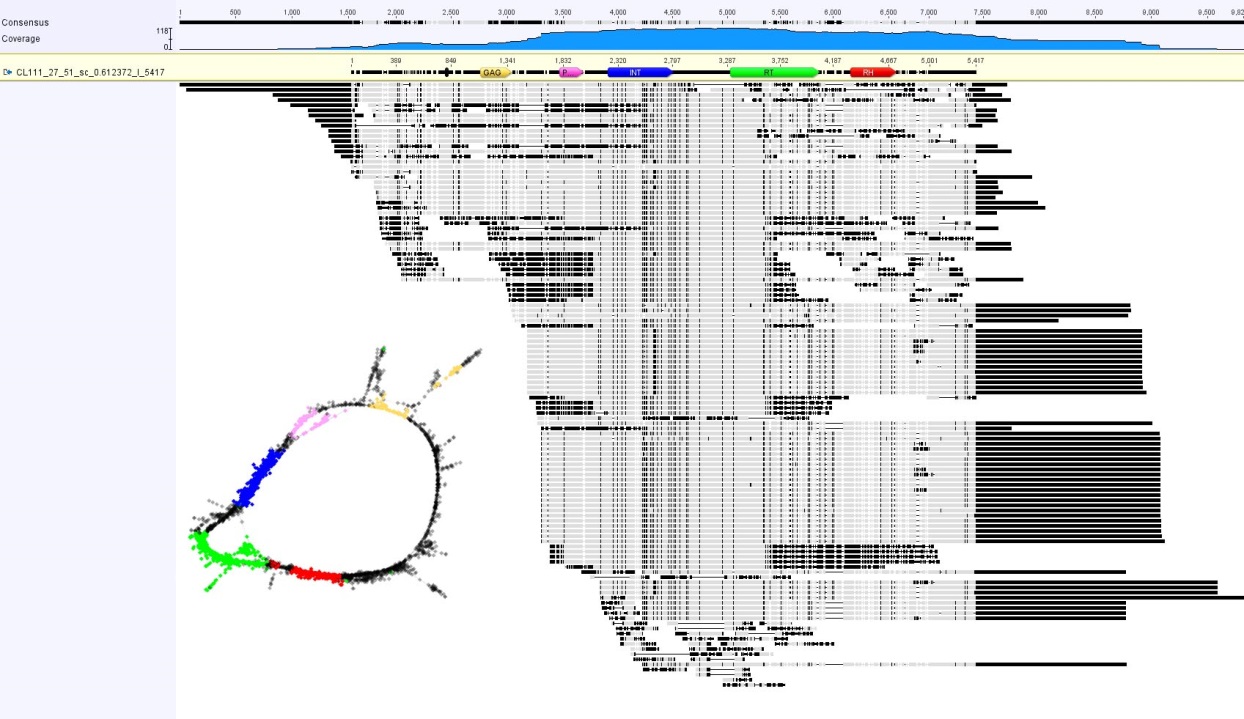


**Figure S3.** This is an alignment of contigs from cluster CL111 (Ty1/copia/TAR). The graph of the cluster is in the bottom left part. Aligned regions are depicted in grey. Unaligned sequences or mismatches are in black. Regions possesing coding domains are depicted in colours (GAG—yellow; PROT—pink; INT—blue; RT—green; and RH—red).
