# Supplementary material for: Comparative Dissection of Three Giant Genomes: Allium cepa, Allium sativum, and Allium ursinum
Source: Int J Mol Sci. 2019 Feb 9;20(3):733. doi: 10.3390/ijms20030733 (PMC6387171; doi:10.3390/ijms20030733)
Supplement: Supplementary file 1 [file ijms-20-00733-s001.zip › 5.ijms-430914-S/suppl_figure/Figure_S4.docx]

**
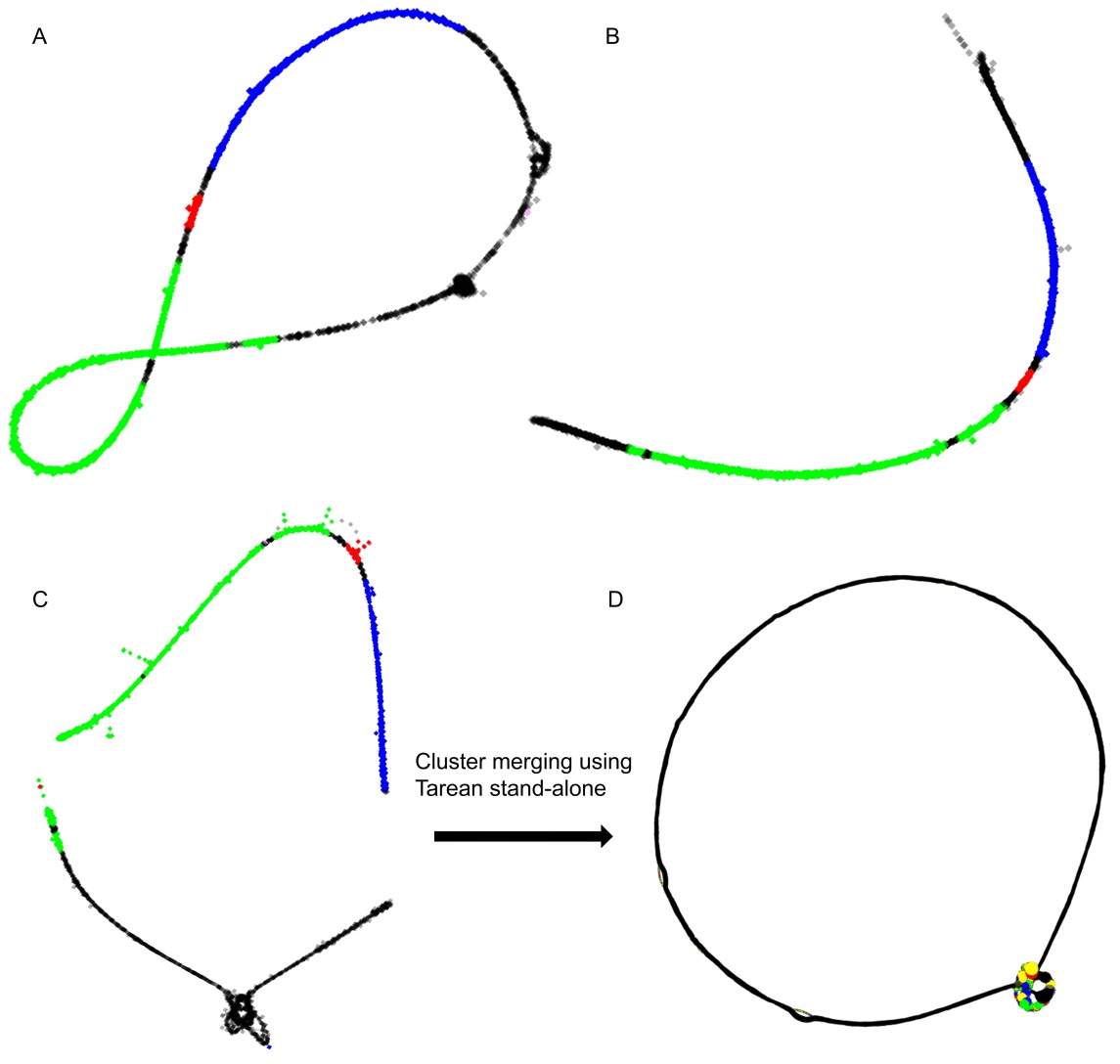
**

**Figure S4.** 45S rDNA clusters. 25S rDNA is in green; 5.8S rDNA in red; and 18S rDNA in blue. Intergenic and internal transcribed spacers are in black. (**A**) *A. sativum* CL57; (**B**) *A. ursinum* CL91 has not provided circular shape and any connection to other clusters through mates was not observed; (**C**) *A. cepa* CL82 and CL87 can be merged into a circular supercluster or graph using Tarean stand-alone (**D**).
