# Supplementary material for: Comparative Dissection of Three Giant Genomes: Allium cepa, Allium sativum, and Allium ursinum
Source: Int J Mol Sci. 2019 Feb 9;20(3):733. doi: 10.3390/ijms20030733 (PMC6387171; doi:10.3390/ijms20030733)
Supplement: Supplementary file 1 [file ijms-20-00733-s001.zip › 5.ijms-430914-S/suppl_figure/Figure_S5.docx]

**
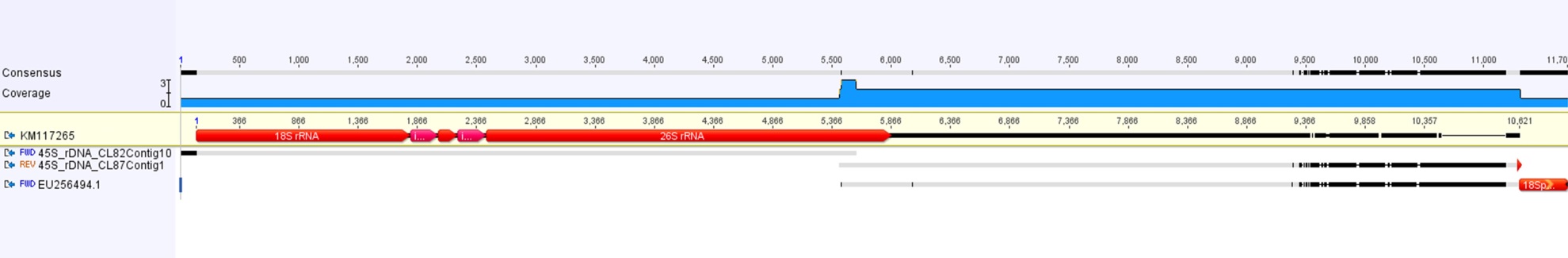
**

**Figure S5.** This is an alignment of contigs from clusters with 45S rDNA from *A. cepa.* 18S, 5.8S, 25S rDNA, and short intergenic spacers are annotated in red. The spacer between 25S and 18S are not annotated. Matching regions are in grey and mismatches in black. The sequence form CL87Contig1 covers this long spacer region and is in congruency with the previously cloned sequence (Genbank: EU256494).
