# Supplementary material for: Comparative Dissection of Three Giant Genomes: Allium cepa, Allium sativum, and Allium ursinum
Source: Int J Mol Sci. 2019 Feb 9;20(3):733. doi: 10.3390/ijms20030733 (PMC6387171; doi:10.3390/ijms20030733)
Supplement: Supplementary file 1 [file ijms-20-00733-s001.zip › 5.ijms-430914-S/suppl_figure/Figure_S6.docx]

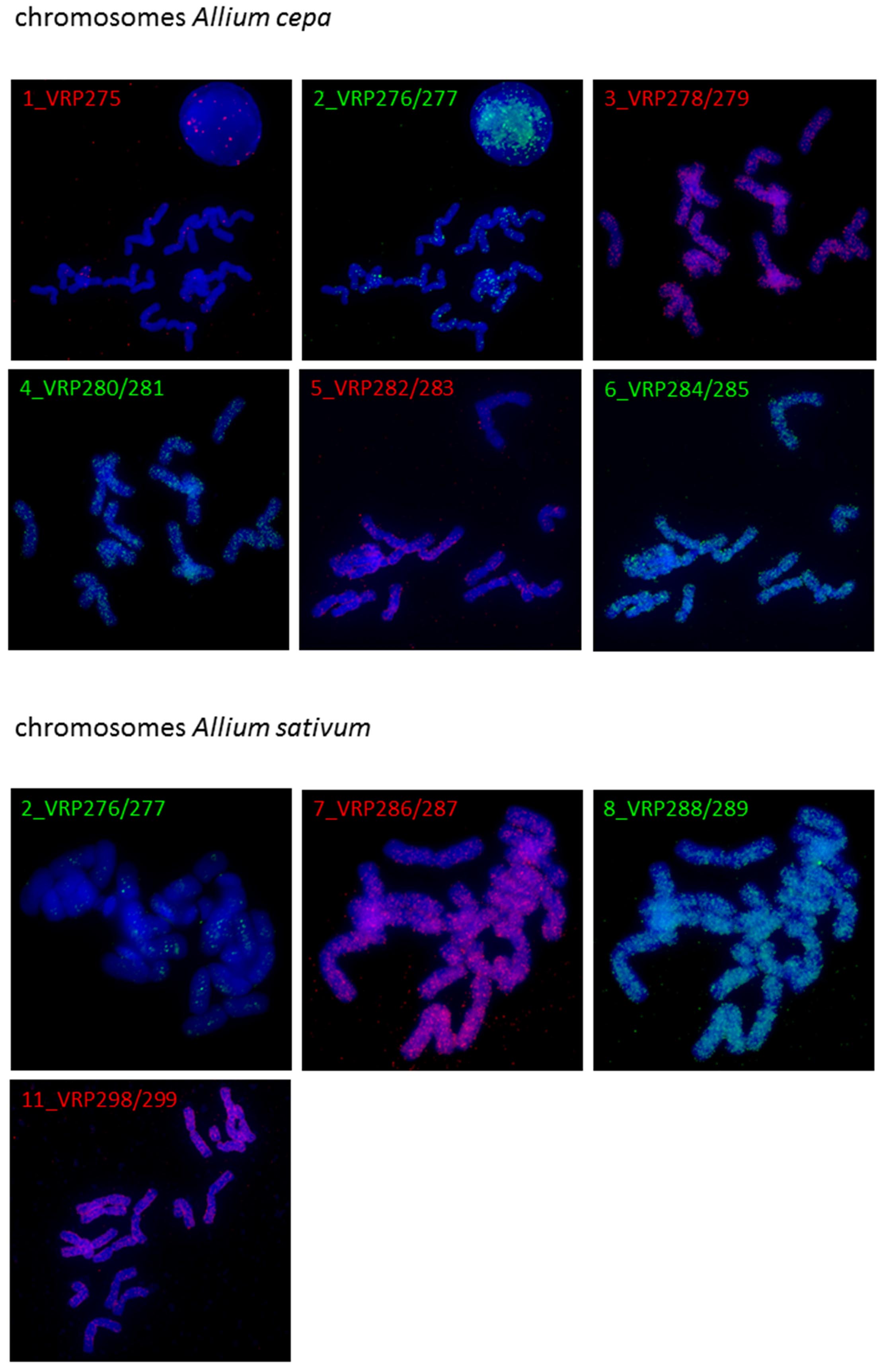


**Figure S6.** FISH experiment with short tandem motifs 1–6 on chromosomes of *A. cepa* (upper part) and 2, 7, 8, and 11 on chromosomes of *A. sativum*. The specific probe signals are in red and green; DAPI staining in blue. The name of probe (upper left corner of each picure) is a combination of number of selected motif (Table 4) and oligonucleotides used for duplex preparation and labelling (Table S9). Experiments without detected signal on chromosomes of any species are not shown (e.g., motifs number 9, 10, and 12).
